# Advanced urothelial carcinoma treated with enfortumab vedotin and pembrolizumab: a path to cure?

**DOI:** 10.1093/oncolo/oyaf259

**Published:** 2025-08-19

**Authors:** Albert Jang, Nikita Tripathi, Sree M Lanka, Hamsa L S Kumar, Prateek Jain, Adam Calaway, Jonathan E Shoag, Randy Vince, Prateek Mendiratta, Santosh Rao, Jorge A Garcia, Jason R Brown, Vadim S Koshkin, Matthew T Campbell, Parminder Singh, Pedro C Barata

**Affiliations:** Division of Solid Tumor Oncology, Department of Medicine, University Hospitals Cleveland Medical Center, Case Western Reserve University School of Medicine, Cleveland, OH 44106, United States; Deming Department of Medicine, Tulane University School of Medicine, New Orleans, LA 70112, United States; Division of Hematology and Medical Oncology, Department of Internal Medicine, Mayo Clinic, Phoenix, AZ 85054, United States; Deming Department of Medicine, Tulane University School of Medicine, New Orleans, LA 70112, United States; Division of Solid Tumor Oncology, Department of Medicine, University Hospitals Cleveland Medical Center, Case Western Reserve University School of Medicine, Cleveland, OH 44106, United States; Division of Hematology and Medical Oncology, Department of Internal Medicine, Mayo Clinic, Phoenix, AZ 85054, United States; Department of Urology, University Hospitals Cleveland Medical Center, Case Western Reserve University School of Medicine, Cleveland, OH 44106, United States; Department of Urology, University Hospitals Cleveland Medical Center, Case Western Reserve University School of Medicine, Cleveland, OH 44106, United States; Department of Urology, University Hospitals Cleveland Medical Center, Case Western Reserve University School of Medicine, Cleveland, OH 44106, United States; Division of Solid Tumor Oncology, Department of Medicine, University Hospitals Cleveland Medical Center, Case Western Reserve University School of Medicine, Cleveland, OH 44106, United States; Division of Solid Tumor Oncology, Department of Medicine, University Hospitals Cleveland Medical Center, Case Western Reserve University School of Medicine, Cleveland, OH 44106, United States; Division of Solid Tumor Oncology, Department of Medicine, University Hospitals Cleveland Medical Center, Case Western Reserve University School of Medicine, Cleveland, OH 44106, United States; Division of Solid Tumor Oncology, Department of Medicine, University Hospitals Cleveland Medical Center, Case Western Reserve University School of Medicine, Cleveland, OH 44106, United States; Division of Hematology and Oncology, Department of Medicine, Hellen Diller Family Cancer Center, University of California San Francisco, San Francisco, CA 94158, United States; Department of Genitourinary Medical Oncology, The University of Texas MD Anderson Cancer Center, Houston, TX 77030, United States; Division of Hematology and Medical Oncology, Department of Internal Medicine, Mayo Clinic, Phoenix, AZ 85054, United States; Division of Solid Tumor Oncology, Department of Medicine, University Hospitals Cleveland Medical Center, Case Western Reserve University School of Medicine, Cleveland, OH 44106, United States; Deming Department of Medicine, Tulane University School of Medicine, New Orleans, LA 70112, United States

**Keywords:** bladder cancer, immunoconjugate, immune checkpoint inhibitor, cystectomy

## Abstract

The combination of enfortumab vedotin and pembrolizumab (EVP) has recently revolutionized care for patients with previously untreated locally advanced and metastatic urothelial carcinoma. However, predictive biomarkers of response, the optimal duration of EVP for patients who respond well to therapy, and the role of circulating tumor DNA to monitor treatment response have not yet been fully elucidated. In this molecular tumor board, we summarize 6 patients from 4 different academic institutions with unresectable or oligometastatic urothelial carcinoma who achieved long-term remission on surveillance after a short duration of EVP treatment that resulted in complete response on follow-up imaging followed by consolidative radical cystectomy. We discuss potential predictive biomarkers for patients with exceptional response to EVP. These findings highlight the need to determine an optimal duration of EVP to maximize efficacy while minimizing adverse events. We aim to further define and explore the value of local therapy with consolidative cystectomy in the setting of unresectable, locally advanced, oligometastatic disease, or those with metastatic disease who achieve complete response of disease outside of the bladder. Identifying this subset of patients who may be candidates for this type of multidisciplinary management may result in durable treatment-free remission.

Key Points

- The frontline combination of enfortumab vedotin and pembrolizumab followed by consolidative radical cystectomy may offer durable treatment-free remission for patients with unresectable locally advanced or oligometastatic urothelial carcinoma with exceptional radiographic response to the frontline systemic therapy.
- Serial longitudinal monitoring of circulating tumor DNA may help identify patients with recurrence prior to radiographic progression. The role of ctDNA in guiding systemic treatment de-escalation and escalation decisions should be explored prospectively.

## Introduction

Immuno-oncology (IO)-based regimens and antibody-drug conjugates (ADCs) have revolutionized the treatment of solid tumors, including urothelial carcinoma (UC). Recently, the combination of enfortumab vedotin (EV), an ADC targeting nectin-4, and pembrolizumab (EVP) has demonstrated superior overall response rates, progression-free survival (PFS), and overall survival (OS) compared to traditional platinum-based chemotherapy in the EV-302 phase 3 trial, establishing EVP as the new standard of care for previously untreated aUC.^1^

Despite the promising data supporting EVP in the metastatic setting, the role of consolidative cystectomy remains uncertain. While cystectomy after chemotherapy is known to benefit a subset of patients with locally advanced or oligometastatic disease,^2^ its role in the context of EVP therapy has not been defined. In this molecular tumor board, we present a summary of 6 patients with previously untreated aUC who received frontline EVP followed by consolidative cystectomy, achieving exceptional outcomes.

## Methods

Six patients from 4 academic cancer centers were pooled together for this analysis. Patients had to have either locally advanced surgically unresectable or oligometastatic disease prior to starting systemic therapy leading to a complete response on imaging, with radical cystectomy afterwards resulting in a pathologic complete response. Baseline demographics, next-generation sequencing (NGS), PD-L1 data based on DAKO 22C3, and quantitative levels of circulating tumor DNA (ctDNA) were collected, if available. The timing of imaging and ctDNA assessment was determined by the oncologist’s discretion at the different sites.

## Results

There were 6 patients (2 male, 4 female) identified from 4 institutions with urothelial cancer in the bladder (Table 1). At the time of diagnosis, 2 patients had stage IIIA UC, 3 patients had stage IVA UC, and one patient was stage IVB UC with an isolated distant metastasis to the pubic bone. At the start of EVP, the median age was 71 years (range 59-78 years). Patients received a median of 6 cycles (range 4-7 cycles) of EVP over a median duration of 4.2 months (range 2.6-4.6 months) before all patients had a complete response on restaging scans. Afterwards, all 6 patients had radical cystectomy resulting in pathological complete response (ypT0N0, pCR). All patients were then placed on surveillance, without evidence of recurrence at the time of data cutoff (median 14 months, range 3-29 months).

**Table 1.** Baseline demographics, treatment information, and follow-up for the 6 patients described in this report.

Case no.	Age at start of systemic therapy	Gender	Tumor histology	Stage	Next-generation sequencing platform	Pathogenic and likely pathogenic alterations	PD-L1 CPS	Tumor mutational burden (mut/Mb)	Sites of metastatic disease prior to EVP	Duration of EVP (months)	Cycles of EVP	Time from initiation of EVP to radical cystectomy (months)	Time since radical cystectomy to last follow-up (months)	Use of ctDNA monitoring after cystectomy
1	73	F	High grade papillary	T2bN0M1b (IVB)	Caris	*ATM, KDM6A, KRAS, TERT* promoter*, TP53, RUNX1*	20	15	Right inferior pubic ramus	4.4	6	5.8	29	Yes
2	66	F	High grade papillary	T4aN2M0 (IIIA)	Caris	*BAP1, FGFR3, JAK2, KDM6A, PIK3CA, STAG2, TERT* promoter	2	12	None	2.6	4	3.7	18	Yes
3	59	M	High grade papillary with plasmacytoid and signet ring features	T4bN1M0 (IVA)	N/A	N/A	N/A	N/A	Rectal invasion	4.6	6	6.5	15	No
4	69	M	High grade papillary	T4bN0M0 (IVA)	Caris	*TSC1, MLH1, CREBBP, CDKN1A*	N/A	5.66	Invasion into the obturator, levator, anorectal junction, right distal ureter	4.2	6	5.6	12	Yes
5	78	M	High-grade papillary with keratinizing squamous differentiation	T4aN1M0 (IIIA)	N/A	N/A	N/A	N/A	Right iliac lymph node	3.9	4	3.9	7	No
6	72	M	Plasmacytoid	T4bNxM0 (IVA)	BostonGene	*ARID1A, PIK3CA, CDH1, CDKN1A, TERT* promoter*, TP53*	25	7	Rectal invasion	4.1	7	5.8	3	Yes

All patients had their primary tumor sites in the bladder. All patients remain alive with no evidence of disease at the time of data cutoff.

Four out of 6 patients had NGS performed on the primary bladder tumor (3 using the Caris platform and one using the BostonGene platform). Three patients had *TERT* promoter mutations. Three patients had PD-L1 combined positive score (CPS) available, with a range of 2-25. Four patients had tumor mutational burden (TMB) available, with a range of 5.66-15 mut/Mb. Four patients had longitudinal ctDNA assessment to monitor for recurrence, with all having persistently negative ctDNA after cystectomy. None of the patients underwent preoperative ctDNA assessment.

## Discussion

Pathologic downstaging for patients with unresectable and advanced UC with induction EVP followed by consolidative radical cystectomy has recently been reported.^3^ To the best of our knowledge, this molecular board is the first to describe in detail 6 patients with unresectable or oligometastatic UC who achieved durable remission following treatment with EVP and consolidative radical cystectomy. All patients experienced a pCR, which is ongoing at the time of the last follow-up. The pCR to EVP regimen, the absence of disease in postoperative restaging scans, and no minimal residual disease detected in the 4 cases with ctDNA assessment, supported the decision to keep them off systemic therapy following cystectomy. These findings continue to support the activity of EVP and the need to further define and explore the value of consolidative cystectomy in the setting of unresectable, oligometastatic disease, or those with metastatic disease who achieve complete response of disease outside of the bladder.

Oligometastatic UC is generally defined as having 3-5 sites of metastasis.^4^^,^^5^ There is little consensus in management, although the combination of systemic therapy with local therapy to primary tumor and metastatic sites has been tried.^4^ The patient with oligometastatic disease involving one single bone metastasis successfully received consolidative metastasis-directed radiation therapy after 4 cycles of EVP, highlighting the potential role for multimodality treatment in oligometastatic UC.

Pathologic downstaging rates in patients with UC treated with chemotherapy or IO therapies are variable, with recent studies showing pCR rates of 31%-50%.^6^^,^^7^ Further, a pathologic downstaging of 50% was observed with EV monotherapy including 36.4% with pCR in the EV-103 cohort H.^8^ Definitive data on the impact of consolidative cystectomy in the setting of EVP remains undefined. In our case series, EVP induced a pCR in all cases. However, the additional benefit derived from cystectomy in patients resulting in pCR remains unclear. Cystectomy may more likely benefit patients with potential resistant disease remaining in their primary tumor after EVP, which suggests the importance of determining the optimal approach and timing of consolidative procedures. Predictive biomarkers for EVP are warranted.

IO therapies have been widely used in several malignancies over the past decade. Biomarkers such as PD-L1 expression and TMB have been associated with response to IO therapies,^9^ but they remain unreliable as true predictive biomarkers. Three cases revealed either high PD-L1 expression (CPS ≥ 10) or elevated TMB (≥10 mut/Mb), highlighting a potential association with exceptional outcomes, although a larger sample size is needed to assess these associations in the context of treatment with EVP. In EV-302, subanalysis of PD-L1 expression (categorized as CPS ≥ 10 or < 10) did not appear to influence treatment response to EVP.^10^

There were 4 patients with NGS on tumor tissue, with 3 patients noted to have a *TERT* promoter mutation. This is one of the most common mutations in aUC,^11^ and the presence of this mutation suggests a worse prognosis.^12^ The data regarding whether the *TERT* promoter mutation is associated with improved outcomes with IO therapies is mixed. Although the presence of a *TERT* promoter mutation was found to be an independent predictor of improved PFS (HR 0.38, *P* = .012) and OS (HR 0.32, *P* = .037) in one retrospective study of 119 patients with aUC for patients treated with IO,^13^ another study of 113 patients with aUC did not find any significant differences in PFS or OS regarding the mutation.^14^ The UNITE real-world database evaluating genomic biomarkers of response to EVP also suggested a lack of association of *TERT* promoter mutations with outcomes.^15^

No confirmed biomarkers are established for EV either. Nectin-4 has been explored as a potential biomarker. Decreased surface expression of nectin-4 of tumor cells may predict resistance to EV in the metastatic setting.^16^ Amplification of the *NECTIN4* gene was also found to be predictive of tumor response to EV and long-term survival for patients with aUC.^17^ In an exploratory analysis of the EV-302 trial for patients with tissue available for nectin-4 and PD-L1 evaluation, all patients benefited regardless of scores over chemotherapy, but patients with a tumor nectin-4 *H*-score ≥275 appeared to have a better median PFS and median OS over patients with tumor nectin-4 *H*-score <275.^10^ Therefore, it is less likely that nectin-4 expression by itself would serve as an adequate predictive biomarker.

Because of the current uncertainty of reliable predictive biomarkers of EVP, another method of monitoring for treatment response is warranted. Circulating tumor DNA (ctDNA) is emerging as a sensitive tool for detecting minimal residual disease and monitoring response in UC.^18^ We have previously shown high concordance rates of ctDNA with CT scans to monitor response to IO in patients with metastatic cancer including UC.^19^ In this current series, undetectable ctDNA after systemic therapy and cystectomy was associated with prolonged remission. These findings support the potential role of ctDNA in monitoring disease and guiding therapy discontinuation. Given the risks associated with prolonged systemic therapy, determining an optimal duration of EVP treatment in patients with a complete response is crucial. Prospective trials evaluating the validity of ctDNA monitoring in this setting are crucial.

Multidisciplinary care for patients with locally advanced and metastatic disease receiving EV with or without pembrolizumab is currently being evaluated in trials. CAST-AI (NCT06764095) is a phase 4 trial evaluating patients who have no progression on EVP after 4 cycles to undergo surgical resection of the primary tumor. CONSOLIDATE (NCT06434350) is a phase 1/2 trial evaluating radiation in combination with EV for muscle-invasive bladder cancer. Both trials will also incorporate liquid biopsy methods to help monitor disease recurrence and progression.

In conclusion, this molecular tumor board presents 6 patients with unresectable locally advanced or oligometastatic UC who achieved long-term remission following treatment with EVP and then consolidative radical cystectomy. These cases underscore the potential for curative outcomes with EVP followed by consolidative cystectomy in select patients with locally advanced or oligometastatic UC. Predictive biomarkers for exceptional responses remain unclear. Further study of the biological underpinnings of those cancers with exceptional response, the role of ctDNA in treatment de-escalation or escalation, and composite biomarkers will be crucial to optimizing multimodality therapy and systemic therapy duration for patients.

## Data Availability

The data underlying this article will be shared on reasonable request to the corresponding author.
